# *Hearts in Action*: supporting precarious workers in Arizona, a community-based participatory research approach

**DOI:** 10.3389/fpubh.2026.1736253

**Published:** 2026-02-26

**Authors:** Dulce J. Jiménez, Alexandra Olin, Shefali Milczarek-Desai, Michael Anastario, Alexandra E. Samarron Longorio, Dolores Encinas, Patricia Wilcox, Floribella Redondo-Martinez, Jill Guernsey de Zapien, Samantha Sabo

**Affiliations:** 1 Center for Community Health and Engaged Research (CHER), Northern Arizona University, Flagstaff, AZ, United States; 2 James E. Rogers College of Law, University of Arizona, Tucson, AZ, United States; 3 School of Nutritional Sciences and Wellness, University of Arizona, Tucson, AZ, United States; 4 Pima County Health Department, Tucson, AZ, United States; 5 Arizona Community Health Workers Association, Yuma, AZ, United States; 6 Zuckerman College of Public Health, University of Arizona, Tucson, AZ, United States

**Keywords:** academic-community partnership, community health workers, community-based participatory research, mixed methods, precarious work, occupational health, immigrant health, Latino/es

## Abstract

**Introduction:**

Precarious low-wage work, marked by instability, limited protections, and high occupational risk, disproportionately affects Latine immigrant workers, contributing to chronic stress, poor health, and cardiovascular risk. In Arizona, sectors such as agriculture, hospitality, and domestic services expose workers to compounded work- and non-work-related stressors, amplified by right-to-work laws and anti-immigrant policies.

**Methods:**

*Hearts in Action*, developed through the community-based participatory research (CBPR) initiative *Aquí Entre Nos*, is a community-driven intervention designed to address work as a social determinant of health and empower workers to advocate for healthy workplaces. Six bilingual and bicultural community health workers (CHWs) co-led four group-based sessions with 50 Latine precarious workers, integrating a workers’ rights advocacy toolkit with an adapted evidence-based cardiovascular health program.

**Results:**

CHWs guided recruitment, retention, facilitation, and participatory data collection, ensuring cultural relevance and creating safe spaces for candid discussion. Strategic partnerships with community and academic partners and legal experts further strengthened intervention design and delivery. Pre-post surveys and semi-structured group reflections assessed changes in health outcomes, occupational self-efficacy, and perceptions about workplace wellbeing and advocacy for change in the labor conditions.

**Discussion:**

Findings underscore CHWs’ critical role in fostering trust, amplifying community voices, and supporting participant empowerment. Hearts in Action offers evidence of feasibility for a CHW-led and worker-centered approach to improve workplace conditions and worker health, offering a model for integrating community perspectives, legal expertise, and public health strategies to promote wellbeing, advocacy, and structural change in the context of precarious labor.

## 1 Introduction

Recent labor discourse has increasingly highlighted the precarity of low-wage work and its effects on workers’ health and wellbeing (1). Precarious work is characterized by uncertainty and instability, where employees bear the risks of employment while receiving limited social benefits and protections (2–6). The consequences of precarious work extend beyond workplace insecurity and safety, impacting non-work domains such as individual physical and mental wellbeing, community cohesion, and broader social structures (7).

Emerging epidemiological and ethnographic research has demonstrated the impact of both work-related (8) and non-work-related (9) stressors on precarious workers. Studies indicate that precarious workers experience numerous adverse health outcomes, such as cardiovascular disease (CVD), psychological distress, a greater number of days in poor physical and mental health, and increased activity limitations (10–12). In the United States, where Latines^1^ constitute the largest immigrant group and a substantial share of the workforce, it is important to recognize that about 40% of precarious workers are immigrants, who may experience additional stressors that worsen health disparities (13).

The present work focuses on specific occupations within this workforce in Arizona, including farmworkers, hotel housekeepers, and domestic cleaners (self-employed or otherwise). These workers typically belong to multiple social groups that face excessive occupational risks, including immigrants, migrants, people of color, women, and low-income workers (8). Job stressors such as job strain, work pressure, worker rights violations, employer retaliation (14, 15), co-worker conflict, musculoskeletal pain as a result of repetitive motion, and exposure to chemicals (9, 16–20) are aggravated by non-work stress factors such as socioeconomic status, immigration status, housing conditions, discrimination, and work-life conflict (10, 11, 21). Together, they create a “double jeopardy” scenario that contributes to health disparities, including chronic stress and CVD (22–24). Additionally, limited legal protection and lack of occupational health and safety laws may worsen these challenges, further contributing to the vulnerability of these workers.

The main objective of this study was to develop and pilot a brief intervention led by CHWs to address work as a social determinant of health, safety, and quality of life among 50 Latine precarious workers in Arizona. This manuscript is intentionally focused on describing the CHW-led intervention and reporting findings derived from the quantitative and qualitative components of the study (i.e., pre-post survey, observations, and group reflections), with particular emphasis on participants’ experiences and an evaluation of primary (self-rated overall health), secondary (physical and mental health measures), and tertiary (occupational self-efficacy) outcomes.

## 2 Methods

### 2.1 Intervention development

*Hearts in Action* is a community-driven intervention that emerged from *Aquí Entre Nos*, implemented through a partnership between Northern Arizona University’s Center for Community Health and Engaged Research and the Arizona Community Health Worker Association (AzCHOW) as part of the NIH-funded Arizona Community Engagement Alliance (AZ-CEAL). Established in 2001, AzCHOW serves as a professional association for CHWs, supporting capacity-building, collaboration, resource sharing, and workforce advocacy. The broader AZ-CEAL initiative aims to partner with communities and community-based organizations to identify and implement effective engagement and outreach practices that communicate trustworthy, science-based information to populations experiencing health disparities.

*Aquí Entre Nos: Juntos Por la Salud (Just Between Us: United for Health)* (herein referred to as *Aquí Entre Nos*) is a collaborative initiative that confronts the challenges faced by Arizona’s precarious workers through a partnership between university and community stakeholders, including workers themselves. The initiative seeks to enhance social connections and build capacity within communities engaged in precarious employment across Arizona. Grounded in principles of community-based participatory research (CBPR), *Aquí Entre Nos* centers the community’s voices and experiences in all efforts to drive meaningful change (25) and positions community health workers (CHWs) or *promotores de salud* (herein referred to as CHWs) as trusted leaders and key agents of advocacy, mobilization, and systemic transformation.

As frontline public health workers, CHWs have a unique understanding of their communities; they share the culture, language, and lived experiences of the clients and therefore serve a vital role in addressing negative medical and social determinants of health (SDoH) among marginalized populations (26, 27). This study adopts a comprehensive approach, addressing both the structural, population-level factors that influence health (i.e., SDoH, such as discrimination, education and employment opportunities, and access to healthcare, housing, and transportation) and their specific, individual-level manifestations (i.e., health-related social needs, such as unstable housing, food insecurity, and interpersonal safety concerns).

As an extension of *Aquí Entre Nos, Hearts in Action* is a pilot program focused on workers’ health, safety, and quality of life program that was co-designed with precarious worker community members and academic partners. The program emphasized the integration of public health and legal strategies to address systemic barriers and enhance the capacity of marginalized communities. Its objectives included raising awareness of the connection between workplace conditions and health, empowering workers to advocate for improved labor conditions, and ultimately improve health outcomes related to job strain—such as common CVD risk factors—by addressing both work-related and broader social stressors.

### 2.2 Intervention description

**Figure 1:**
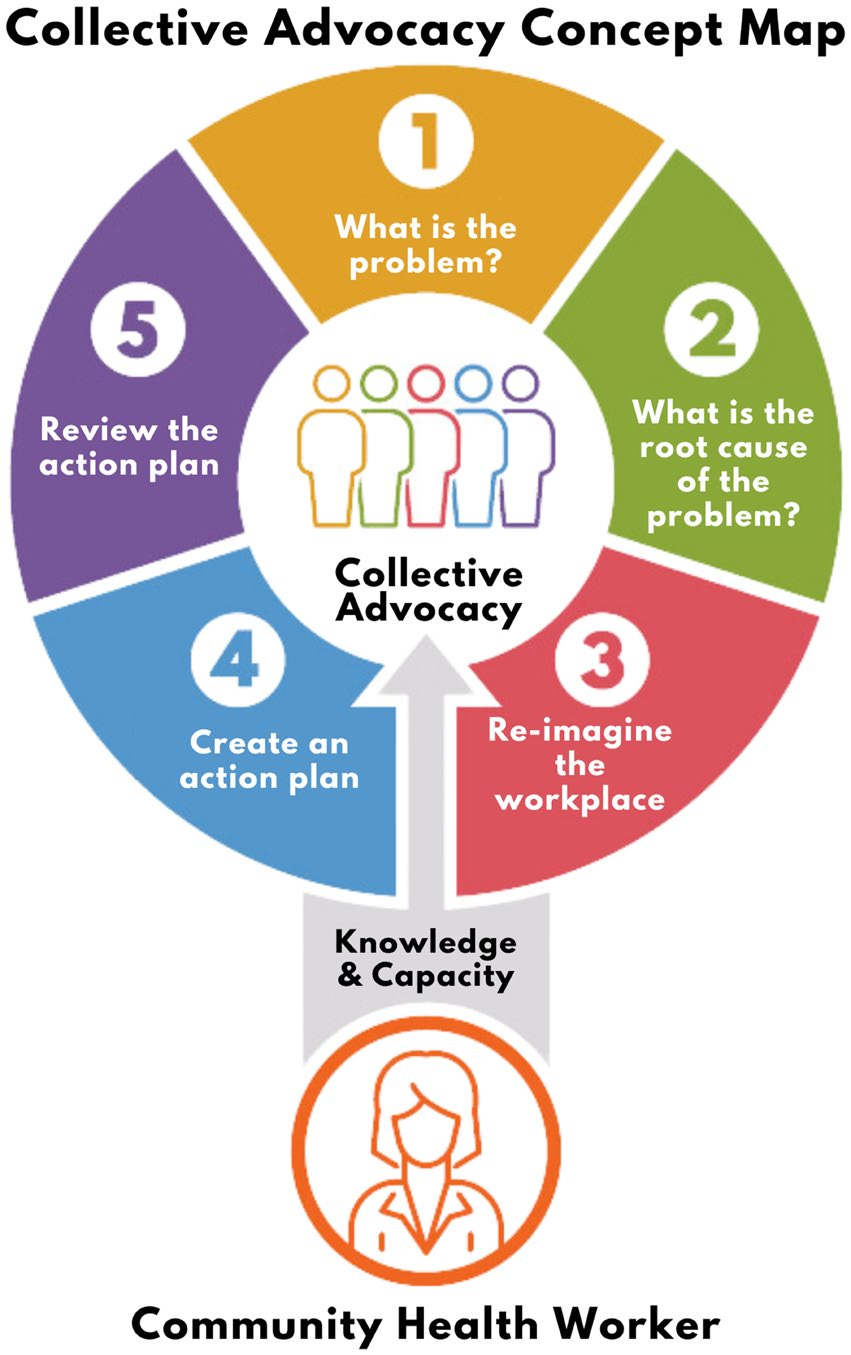
The five step advocacy plan to improve workplace health.

*Hearts in Action* integrated a workers’ rights toolkit that guides participants through a five-step advocacy plan to improve workplace conditions (see Figure 1). The toolkit was created in 2020 as direct response to the *Aquí Entre Nos* CAB’s urgent call for workers’ rights education amid the COVID-19 pandemic. Collaboratively developed with legal, public health, and community partners, the toolkit provides expertise and resources to support precarious workers in addressing poor workplace conditions Arizona. The intervention was further adapted from the CHW-delivered evidence-based National Heart, Lung, and Blood Institute (NHLBI) program, “Your Heart Your Life” (28), which the team has tailored to regional contexts to address cardiovascular health (29, 30). Hearts in Action modified this program’s foundational CHW facilitation components, such as the participatory introduction, goal setting exercises, and action-planning framework, rather than cardiovascular-specific content. Supplementary Table S1 summarizes the intervention by session topics.

The *Hearts in Action* intervention consists of four, two-hour sessions delivered by CHWs. The sessions are group-based, with 5–10 precarious workers participating in each group. The same group of workers participates in four consecutive sessions together guided by one or two CHWs, depending on group size and CHW capacity. Initially, the intention was to hold one session per week over a month-long period. However, CHWs and workers had the flexibility to choose the day, time, location, and frequency of the sessions. Most sessions were held in a period of two weeks between February and March of 2024, with the groups meeting multiple times per week.

Below is a detailed breakdown of each session.

1. **Session 1**: Introduction to health and labor and work as a SDoH.

- This session involves introductions to the group and the project. At the start of the session, several participants had already consented and completed the baseline survey over the phone or online. Participants who were new to the study were pulled aside by a research staff member to complete informed consent and the survey, while the other participants and CHWs settled in, grabbed refreshments, and talked with each other. Once all participants completed the consent and survey, the CHWs facilitated an ice-breaker activity for the group of workers to get to know each other. The CHWs then provided a brief overview of the research project and aims and participant involvement and incentives. Finally, the CHWs facilitated an activity and group conversation to together reflect on how labor experiences impact worker wellbeing, introducing work as a SDoH.
- Participants are given a take home questionnaire on workplace conditions that impact health and prompted to reflect on their own experiences to be shared with the group in the following session.

1. **Session 2**: *Step 1*. What is the problem? *Step 2*. What is the root cause of the problem?

- Session 2 entails the first two of the five steps to improve workplace health. Participants return to this session having used the questionnaire on workplace conditions as a reflection tool to think about concerns they have related to work and health. The CHWs then ask participants to identify one priority issue they want to focus on and explore why that problem exists. Workers are encouraged to consider the root causes of the problem, emphasizing structural or organizational policies, practices, and norms rather than individual-level behaviors.
- Participants are given a reflection prompt to take home and discuss with the group during the following session. Prompt: “Write 3 characteristics of your workplace that could support your wellbeing. Consider, what would your ideal workplace look like?”

1. **Session 3**: *Step 3*. Re-imagine your workplace. *Step 4*. Create an action plan.

- Session 3 involves steps 3 and 4 of the five steps to improve workplace health. Participants regroup for this session after being asked to engage in a personal reflection to help them think creatively about strategies and solutions to improve the workplace. The CHW guides conversations to identify existing strengths and assets and highlight changes that could be made to create a work environment that is more supportive of worker wellbeing. Then, participants develop an action plan with a timeline and specific steps to take towards creating workplace change. Participants are provided with a printed worksheet for personal use to write down their action plans and reflections.
- Participants are instructed to practice their action plan at home with a trusted family member or friend, or to execute it in the workplace if they feel safe and ready to do so. The CHWs acknowledge that not every person may be able to move forward with their action plan depending on their workplace situation and encourage participants to do what feels right to them.

1. **Session 4**: *Step 5*. Review the progress on the action plan.

- The final session is intended to reflect on both the five steps, including any outcomes of practicing the action plan, and the overall experience participating in the *Hearts in Action* program. Participants are encouraged to share the outcomes of practicing the action plan and brainstorm future action steps, including changes in their own knowledge, perspectives, or attitudes related to workers’ rights. Finally, participants and CHWs together reflect on their overall experiences in the program, including an evaluation of what went well and what could be improved.

### 2.3 Community engagement

A variety of community-grounded strategies were utilized to develop, implement, and evaluate *Hearts in Action*. The approach began with community engagement and strategic partnerships, highlighting the contributions of advisory boards, CHWs, and multi-sector collaborations in guiding the project. CHWs served as co-researchers in intervention design and implementation, contributing to recruitment, facilitation, and participatory activities. The intervention description outlines program content, structure, and delivery, followed by data collection and analysis methods used to assess participant outcomes and intervention effectiveness.

### 2.4 Building strategic partnerships

Building on the community advisory board (CAB), established at the onset of *Aquí Entre Nos*, Latine workers shaped the overall direction of *Hearts in Action*, ensuring that the program was culturally relevant and applicable. The CAB identified workers’ priorities and helped conceptualize the project’s vision and goals grounded in the lived experiences of Arizona workers. Details of this partnerships and engagement strategies are described in Sabo et al. (31).

CHWs and CHW leadership from AzCHOW were also engaged early on, leveraging community knowledge with academic expertise to develop and implement *Hearts in Action*. The six CHWs who delivered the intervention were bilingual, bicultural, and embedded within the communities they served. Their contributions informed community engagement strategies and the intervention’s final design, content, and delivery format. The CHW interventionists underwent training on the SDoH, with particular emphasis on work and its relationship to chronic illness and stress, including cardiovascular disease, to strengthen CHWs’ knowledge, facilitation skills, and confidence in leading open discussions on sensitive and complex topics related to precarious work and health.

Finally, a technical advisory board was formed in partnership with the University of Arizona College of Public Health and College of Law to include public health and legal expertise. Importantly, some members contributed long-term immigration community organizing experience in Arizona, providing grassroots knowledge. Rooted in a shared commitment to justice, these partners contributed to the intervention’s design, implementation, and evaluation and provided legal education and resources to help precarious workers better understand their rights in the workplace.

### 2.5 CHWs as co-researchers

As co-researchers, CHWs shaped every stage from recruitment and retention to data collection, analysis, and dissemination of results. The six CHWs, from across three rural and urban Arizona cities recruited 5–10 precarious workers using purposive word-of-mouth and snowball sampling within their social networks and communities. Unlike standard research recruitment methods that rely on advertising or institutional outreach, CHWs employed personal, trust-based communication grounded in shared lived experiences. Drawing on their own prior experience in precarious labor and knowledge of the local community, they strategically engaged individuals who were often underrepresented in conventional outreach efforts.

Utilizing participatory methods, such as structured group reflections and case studies, the CHWs facilitated discussions where both workers and CHWs shared personal stories, creating a mutual learning environment. During the four sessions, the CHWs guided group conversations with participants about the connection between work and health. Drawing on their own lived experiences as immigrant and Latine individuals who previously engaged in precarious work, the CHWs built strong relationships with participants based in mutual respect, trust, and learning. Additionally, the CHWs used the legal knowledge from the workers’ rights toolkit developed with law partners to discuss strategies informed by law to change workplace conditions. All sessions were held in Spanish with Latine workers and CHWs.

To support retention, CHWs created accommodating environments, adapted to participant needs, and maintained communication through calls, texts, and group chats. During intervention delivery, CHWs established group norms through collaboratively developed community agreements that reflected shared values—such as mutual respect, active listening, compassion, and confidentiality—and made ongoing decisions about communication, session logistics, and group dynamics. Barriers were addressed with culturally and contextually appropriate strategies, including holding sessions in familiar spaces (e.g., churches, homes, community centers, libraries), scheduling at convenient times (e.g., late evenings, Sundays), providing light refreshments, and adjusting session structure based on real-time feedback. For example, although the intervention was originally designed as four weekly sessions over a month, CHWs and participants often condensed the timeline and met multiple times per week to maintain momentum and interest. Remaining flexible in this manner improved recruitment and retention among a hard-to-reach population.

Finally, CHWs contributed to data collection and qualitative analysis, ensuring findings accurately reflected participants’ experiences and translating results into culturally relevant materials for community members and stakeholders. To achieve this, CHWs were brought together via Zoom twice during the active intervention and debriefed with researchers before and after each reflection session to interpret the data.

### 2.6 Data collection and analysis

A pre-post survey and a final semi-structured group reflection were utilized to assess changes in overall health status, work environment perceptions, and self-efficacy to advocate for improved workplace conditions before and after the intervention.

#### 2.6.1 Quantitative data: survey

The survey utilized a single-group pretest-post test design, where each participant’s outcomes were measured before and after the intervention to assess changes. A pre-post survey including close-ended and open-ended questions was administered to evaluate the outcomes of the pilot intervention on self-reported overall health status (primary outcome), physical and mental health measures (secondary outcomes), and occupational self-efficacy (tertiary outcome) among a sample of 50 participants. All statistical analyses were performed in STATA.

##### 2.6.1.1 Surveys power analysis

To determine statistical power, the following scenario was simulated using self-reported overall health status values derived from previous pilot research with hotel housekeepers (31), and intra-class correlation coefficient values derived from Dal Grande et al. (32): 50 participants in a closed cohort with data at baseline and follow-up; an average self-reported overall health status at baseline of 3.0 (corresponding to good); 5 CHWs each delivering the intervention to 10 participants; a 0.75-point improvement in self-reported health immediately following the intervention (corresponding to the upper portion of the range from good to very good); and an intraclass correlation coefficient of 0.6. Based on 10,000 replicates on 50 participants observed at baseline and follow-up, 96% power was detected against the null hypothesis of no effect on the primary outcome of self-reported overall health status at the *α* = 0.05 level. However, as a pilot intervention, the sample size is assumed to be small, and more attention is given to the direction of effects detected rather than their statistical significance. Results of this study are intended to inform the design and implementation of a future larger scale intervention.

##### 2.6.1.2 Survey data analyses

Initially, a series of ANOVA tests were conducted to examine bivariate relationships between participant characteristics and primary and secondary outcomes. These analyses aimed to identify potential confounders that could be adjusted for in subsequent models. CHW cluster groups were also treated as potential confounders in the context of this pilot study.

Next, a series of multilevel models was developed to assess changes in the outcomes relative to the intervention. In each multilevel model, a random intercept was included for individuals to account for within-subject correlations, recognizing that repeated measures on the same individuals are likely to be more similar than measures across different individuals. This approach allowed for the estimation of both fixed effects (e.g., intervention effects, clustering by CHW, and potential confounders) and random effects (individual variability).

Three models were run for primary and secondary outcomes. The first model in the sequence assessed the effect of the intervention by comparing pre-intervention and post-intervention periods without adjusting for any additional variables. The second model included the grouping of participants by CHW to control for potential clustering effects within these groups. The third model further adjusted for potential confounders identified through the bivariate analyses, such as gender and other demographic variables.

#### 2.6.2 Qualitative data: field note observations and final group reflections

Researchers attended multiple sessions and took handwritten field notes using an *a priori* observation template created with specific constructs to evaluate the intervention. CHWs co-facilitated semi-structured final reflection sessions with the research team, during which participants discussed intervention outcomes, including changes in knowledge, perspectives, and attitudes toward workers’ rights, and plans for applying what they learned. Leveraging trust and shared experiences in precarious labor, CHWs elicited candid reflections on challenging topics to discuss such as workplace discrimination.

One researcher was present in all the final sessions and audio recorded the final reflections. Audio recordings were transcribed and handwritten notes were typed into digital documents. Handwritten and audio transcripts were entered into ATLAS.ti. Thematic analysis using inductive coding was conducted, where a codebook was developed and refined iteratively throughout the coding process. Two researchers coded the data separately in ATLAS.ti and meet weekly to discuss their findings until consensus was reached on final themes.

CHWs contributed to validating and contextualizing the results through a collaborative member-checking and reflection process. Researchers and CHWs met to assess the accuracy of the findings, explore public health implications for precarious workers, and identify next steps.

## 3 Results

### 3.1 CHW capacity building and research contributions

Co-facilitating reflection sessions enhanced CHWs’ leadership and research capacity by involving them directly in data collection and interpretation, evidenced by their ability to elicit deeper participant reflections and apply research concepts within community contexts (33). By encouraging candid dialogue and contextualizing findings, CHWs helped understand nuanced insights, contributing to the analytical interpretation and cultural validity of data analysis. Their involvement highlighted the critical role of CHWs as co-researchers in strengthening methodological rigor and cultural relevance to inform more equitable research practices. The collaborative approach to data interpretation centered community voices in the findings and fostering shared ownership of the study’s outcomes and future direction.

Engaging CHWs as co-investigators across phases of the research process is a well-documented best practice in health intervention research that enhances study relevance and community engagement. (34). Beyond traditional clinical and community health roles, CHWs are well-positioned to serve as advocates for worker health and safety, leveraging their community ties to address the SDOH and occupational challenges among precarious workers (35). A 2018 systematic review by Swanberg et al. synthesized evidence on CHWs’ contributions to occupational safety and health research, underscoring their potential to support worker-centered interventions and improve workplace health outcomes (33). Together, this literature highlights CHWs as key partners in advancing worker-centered health interventions and provides a strong rationale for the present study.

### 3.2 Survey data

#### 3.2.1 Participant characteristics

Supplementary Table S2 presents the baseline characteristics of the participants relative to primary and secondary outcomes. The sample included a higher proportion of females (68%) compared to males (30%), with 2% self-identifying their gender identity as other. Most participants identified as Hispanic/Latine (86%). The age distribution showed that the largest group of participants were aged 40–49 years (44%), followed by those aged 50–59 years (20%). The majority of participants worked in hotel housekeeping/domestic cleaning (46%), followed by agriculture (32%), the restaurant/food industry (14%), landscaping (6%) and construction (2%). Nearly all participants were born outside of the USA (98%) and spoke a language other than English at home (96%). At baseline, hypertension and cholesterol were the most common issues identified (both at 30%), followed by pre-diabetes and depression (both at 26%), pre-hypertension and diabetes (both at 12%), and cardiovascular disease (6%).

In the bivariate analyses, the primary outcome (self-assessed health) varied significantly in relation to gender identity and clustering CHW (Supplementary Table S2) at baseline. Across primary and secondary outcomes, there was appreciable variation in relation to clustering by CHW, hence this cluster was controlled for as a fixed effect in Model 2. Finally, the secondary outcomes also varied in relation to different participant characteristics, and these were accounted for in Model 3.

#### 3.2.2 Mixed-effects models for primary and secondary outcomes

Supplementary Table S3 shows post-intervention improvements across primary and secondary outcomes, although confidence intervals included the null. For overall self-assessed health, slight improvements were observed in Models 1 and 2 (*β* = −0.2, 95% CI: −0.4 to 0.1), but the association was attenuated after adjustment for potential confounders (*β* = 0.0, 95% CI: −0.4 to 0.4).

Similarly, small improvements were consistently observed in the remaining outcomes (i.e., physical health limiting physical activity, difficulty performing everyday activities, physical or emotional problems limiting social activities, and self-assessed emotional problems), with effect sizes ranging from 0.12 to 0.32 (Supplementary Table S3). However, confidence intervals were wide and crossed the null. Adjustment for clustering by CHW had little effect, while adjustment for potential confounders further weakened associations. The modesty of the effect sizes coupled with large confidence intervals across the primary and secondary outcomes should be interpreted in the context of this being a pilot study.

#### 3.2.3 Tertiary outcome

Supplementary Table S4 presents the results for the tertiary outcome relative to baseline and follow-up. Effects are presented for models including only the post-intervention period as a fixed effect after adjusting for clustering by CHW. All occupational self-efficacy measures moved in a favorable direction post-intervention, except for the item assessing preparedness for a future career, yet confidence intervals included the null.

Across outcomes, the observed effects moved in the hypothesized direction, providing promising results for a future study with a larger sample size and a greater number of observation periods (before, during, and after the intervention).

### 3.3 Qualitative data

*Hearts in Action* intervention engaged 50 precarious workers across three geographic points in Arizona, including two urban cities and one rural community. Participants were employed in precarious work sectors including agriculture, hotel housekeeping, domestic cleaning, and restaurants.

The findings below are based on researcher field observations collected during the intervention and final reflection sessions across all worker groups participating in Hearts in Action. A central aspect of the intervention was to provide a platform to facilitate worker dialogue and solidarity. Results reflect participants’ general lived experiences discussed during sessions, except in the Application of Curriculum section, which captures how participants applied or planned to implement components of the intervention in their lives. Findings are organized into four overarching themes: precarious work challenges; solutions and strategies; support needed; and application of curriculum. All participant quotes were originally in Spanish and translated into English by the research team for inclusion below.

#### 3.3.1 Precarious work challenges

##### 3.3.1.1 Workplace conditions

Participants reported numerous physical and mental health challenges arising from poor work conditions. Stressful environments, extreme temperatures, exposure to hazardous materials, and lack of proper training were common issues. Many workers also described the emotional toll of these conditions, including physical symptoms such as headaches, fainting, and irritability. One participant highlighted the intensity of these experiences: “They have you in slavery, with a foot on your neck,” reflecting a feeling of oppression. Another described the emotional strain as overwhelming, stating, “That stress, that anger is everything. And do not you think it’s not [true], because sometimes it happens to you, sometimes you even feel like crying.”

##### 3.3.1.2 Interpersonal relationships with workplace management

A significant source of workplace stress was the perceived lack of concern for workers’ wellbeing from their management. This was evidenced by the prioritization of operational profits over health (e.g., disregarding sick leave to ensure team staffing or turning off air conditioning to save costs). Leadership often employed intimidation tactics, creating a pervasive atmosphere of fear in which participants felt vulnerable and afraid, as reflected in one account: “I lived in fear.”

##### 3.3.1.3 Unfair treatment, discrimination, and workplace injustices

Participants shared numerous instances of discrimination and unfair treatment in the workplace based on their intersecting identities and social groups (e.g., race, ethnicity, immigration status, gender, age) as well as personal relationships with leadership. These discriminatory practices frequently resulted in specific harms such as wage theft, verbal abuse, and favoritism, which were often based on participants’ ethnicity, immigration status, nationality, and language. For instance, one participant recalled being paid less than the minimum wage due to their supposed “undocumented” status, despite having legal documents (i.e., management incorrectly assumed they were undocumented). Furthermore, some reported incidents of sexual harassment and discrimination based on gender and sexual orientation that led to unfair treatment in their jobs, such as being assigned to certain tasks or workspaces or given different workloads.

##### 3.3.1.4 Feeling unappreciated

A pervasive theme was the feeling of being undervalued and easily replaceable. Many participants noted that their hard work went unrecognized and they were reprimanded for minor mistakes, making them feel invisible and unsupported. As one worker pointed out, “We are not slaves; we are workers, and we have rights,” underscoring the need for recognition and respect.

##### 3.3.1.5 Fear of retaliation

The fear of retaliation was a key factor preventing workers from voicing their concerns. Many participants were concerned that speaking out about issues could jeopardize their job security, particularly those with precarious immigration status who feared further mistreatment. This fear was compounded by economic instability, making it difficult for workers to advocate for their rights.

##### 3.3.1.6 Language barriers

Language differences were another significant barrier. Participants reported that language difficulties, especially when communicating with supervisors or clients, contributed to their stress and made it harder to advocate for better conditions. The lack of proficiency in English made workers feel vulnerable in expressing their needs or negotiating terms.

##### 3.3.1.7 Work–family conflict

The long hours and stress of work often impacted workers’ family lives. Participants spoke about the difficulty of balancing work and family responsibilities, leading to feelings of isolation and guilt. One participant noted, “Because here there’s a lot of overtime and they say that people end up working for a long time and then they do not spend time with their family.” They went on to describe how the job strain may spill over into home life, causing stress and feelings of anxiety and depression that further affect personal relationships.

#### 3.3.2 Solutions and strategies for addressing work challenges

Despite these difficulties, participants shared various strategies to mitigate work-related challenges. Each session ended with a small group reflection. The final session asked participants to reflect on the practical application of what they learned, suggested changes to the program, and their overall experience in it. Many reported being self-employed, particularly as domestic cleaners, and exchanged tips on how to protect themselves at work. Common strategies included creating contracts, setting boundaries, and attending small-business owner classes to learn how to better manage their work.

Moreover, participants often responded to each other’s experiences by offering practical solutions for navigating difficult workplace dynamics. These strategies included educating themselves on their rights, establishing personal boundaries, speaking up assertively, and organizing collectively with colleagues. As one participant remarked, “If we do not take action ourselves, nothing is going to change,” emphasizing the importance of self-advocacy. Similarly, another individual reported that the intervention helped the group activate to develop a plan and create change in their workplace conditions, “You came to wake us up.”

A different worker shared how participating in the intervention helped them learn about potential strategies to address workplace challenges and feel better prepared to approach their co-workers and find a solution together:

Personally, I felt very good expressing my work-related problems and the stress it usually causes me. The various opinions my colleagues shared helped me realize that we can have several solutions, right? One is to work as a team, meaning that not just one person should know how to operate a machine or something, but we should teach more co-workers so they can do it too, and that will reduce stress for me.

#### 3.3.3 Support needed by precarious workers

Participants expressed a need and a desire for more education and information overall on workers’ rights topics. For instance, they wanted further guidance in creating action plans and strategies, and details regarding their legal rights as employees. This includes more programs like *Hearts in Action* to help workers feel heard, valued, and supported:

That there be more programs like this, because in the 20 years I've been working here, this is the first time I've seen a program like this that helps advise us. In the good sense of the word, that opens our eyes so that, in some way, we don't feel just not alone, but that we feel the support of a program like this helps a lot… That there be more programs like this so that we can have more connection with a coworker, because I have a connection with her [*points to her coworker, who was also a program participant*] because we work in the same hotel, but not in this way. Where we can talk calmly without the pressure that she has to do her job and I have to do mine. However, here we are talking very calmly, but this is a result of this program that exists.

Participants also discussed gaps in the SDoH in their community, such as medical resources, reliable and affordable childcare, and financial support for people who were laid off and are having trouble finding new employment: “It is necessary to have medical access, to have insurance, to have benefits.” Suggestions for resources related to workers’ rights included a community forum with legal experts, a list of accessible lawyers with expertise in labor issues, and wage theft workshops.

One group in a rural farming community concluded that they need an outside person with expertise on laws and workers’ rights to advocate on their behalf and represent them in talking with the leadership of farming companies to make a change (i.e., the town is so small that action from any one individual would place them at great risk of being isolated from the community and not being able to get hired anywhere in town).

#### 3.3.4 Application of curriculum

The intervention equipped participants with valuable tools to improve their work environments. Several participants reported applying their learning in real-life situations, such as initiating conversations with their supervisors about work conditions or mediating conflicts with colleagues. For instance, one housekeeper requested a more balanced schedule, while another effectively handled a dispute with a colleague.

Participants emphasized that they now understood the importance of preparing for workplace advocacy through careful documentation, note-taking, and structured approaches. They also reflected on the connection between work stress and health, noting how labor-related stress can affect home life and relationships, and the value of adjusting attitudes or engaging in proactive conversations to manage stress. Participants discussed their intentions and plans to apply the knowledge and skills gained during the intervention in their daily lives, including sharing this information with others: “I would like to implement all the ideas I learned here in my job, the experiences I had, and be able to carry them out for my own benefit and that of my family.”

## 4 Discussion

*Hearts in Action* emerged as a direct response to the health and labor challenges expressed by Latine precarious workers in Arizona, developed with workers, for workers. Grounded in community-based participatory research, the project was shaped by community concerns about precarious working conditions, discrimination, immigration-related fears, and chronic stress (24, 31). It aimed to recognize and address work as a SDoH and empower precarious workers through information and social support to know their rights to better navigate wellbeing in the workplace (36).

CHW participation transformed the project from a researcher-driven agenda into a community-led process, with CHWs contributing as co-investigators at every step. Engaging CHWs as co-researchers centers community expertise, builds research capacity, and facilitates candid dialogue to produce nuanced insights, enhancing overall study relevance, rigor, and social impact (37, 38). CHWs bridged academic and community perspectives by applying research concepts in ways that resonated with participants and ensured their experiences were authentically represented. This role was especially critical amid heightened anti-immigrant sentiment, enabling open discussion of sensitive issues such as workplace exploitation, immigration-related stress, and health disparities (38). CHWs created a safe space where precarious workers could find solidarity and together, emotionally process previously unacknowledged experiences of workplace injustices and abuse. By fostering trust through a culturally grounded approach, CHWs made the intervention more empowering and impactful for participants.

The stressors faced by precarious workers can be amplified by broader sociopolitical factors such as the practices and policies that govern labor and occupational protection. Arizona’s right-to-work and anti-immigrant policies and rhetoric contribute to a sociopolitical climate that limits workers’ ability to access health and legal resources and advocate for themselves (24). Right-to-work (RTW) laws, which allow employees to opt out of union membership and dues even in unionized workplaces (39), have been linked to adverse health (40) and economic (41) outcomes for workers. These laws limit the power of collective bargaining and contribute to declining unionization rates, which in turn weaken workplace protections and negatively impact worker health and safety (40). Furthermore, RTW laws exacerbate both work-related and non-work-related stressors, creating a double jeopardy effect for precarious workers. Financial instability is elevated as workers in RTW states often earn lower wages and have reduced access to employer-sponsored benefits, making it difficult to secure housing, healthcare, and other essential resources (40–42). The intersection of these financial and occupational challenges may deepen health inequities and perpetuate long-term socioeconomic disparities among precarious workers. Given that union density in RTW states is roughly half that of non-RTW states, precarious workers in states such as Arizona are more likely to live in poverty, lack health insurance, earn less money, and have higher mortality rates compared to states without these laws (31, 41).

These challenges are compounded by Arizona’s anti-immigrant legislative history and persistent sentiment, which create a climate of fear that limits access to resources and services among communities of color and immigrants (43, 44). Policies such as SB 1070, which authorized local law enforcement to verify immigration status and increased the risk of immigration-related penalties, intersect with RTW and anti-union laws, heightening Latine precarious workers vulnerabilities (45, 46). In turn, this sociopolitical environment facilitates employer practices that exploit and contribute to workers’ lack of knowledge about their rights. This is evident in the prevalence of worker misclassification, where some precarious workers, particularly in the cleaning industry, believe they are self-employed (i.e., independent contractors) when they are actually misclassified employees legally entitled to fundamental rights. Furthermore, farmworkers often lack clarity regarding the identity of their real employer (e.g., the farmer versus a contractor), intensifying their employment precarity.

Overall, *Hearts in Action* underscores the value of CBPR in addressing health disparities among precarious workers. By engaging CHWs and adopting participatory methodologies, this project identified structural barriers and provided tools and knowledge for workers to advocate for themselves in the workplace. Community-university and multi-sector partnerships were intentionally cultivated to implement CBPR principles such as co-learning, shared leadership, and action-oriented outcomes. Leveraging community knowledge with academic and legal support allowed for an intervention that was responsive to immediate needs while laying the groundwork for broader systemic change. In Arizona, where political and economic factors significantly constrain precarious worker rights and access to resources, partnerships between community organizations, academic institutions, and other local stakeholders are needed to effect meaningful change.

Hearts in Action provides evidence that a CHW-led, worker-centered intervention can feasibly support precarious workers in gaining the knowledge, tools, and confidence to improve their workplace conditions and health.

## 5 Conclusion

*Hearts in Action* demonstrates the transformative potential of community-driven research in addressing health disparities among precarious workers. The participatory methods employed enhanced precarious workers’ knowledge and advocacy skills while also creating a supportive community where workers could share their experiences and learn from each other. By addressing work as a SDoH, *Hearts in Action* contributes to a broader commitment to promote fairness, improve working conditions, and enhance the overall health of precarious workers in Arizona.

## Data Availability

The original contributions presented in the study are included in the article/Supplementary material, further inquiries can be directed to the corresponding author/s.

## Footnotes

1 Note on terminology: The term “Latine” reflects the evolving language practices within communities. 40 It is an alternative to Latino, Latina, or Latinx and promotes respect for cultural and individual 41 identities.

## References

[1] Milczarek-Desai S. (2026). Moving Beyond Worker Rights to Worker Justice. Tex AM R. 14.

[2] Hewison K. Precarious work In: Edgell SGH, editor. The Sage Handbook of the Sociology of Work and Employment. Thousand Oaks, CA: Sage (2016). 428–43.

[3] Kalleberg AL. Nonstandard employment relations: part-time, temporary and contract work. Annu Rev Sociol. (2000) 26:341–65. doi: 10.1146/annurev.soc.26.1.341

[4] Kalleberg AL Hewison K. Precarious work and the challenge for Asia. Am Behav Sci. (2013) 57:271–88. doi: 10.1177/0002764212466238

[5] Rodgers G Rodgers J. Precarious work in Western Europe: The state of the debate. In: Precarious Jobs in Labour Market Regulation: The Growth of Atypical Employment in Western Europe [Internet]. Geneva: International Institute for Labour Studies. (1989) p. 1–16. Available online at: https://ilo.org/global/publications/

[6] Vosko L. Introduction. In: Managing the Margins: Gender, Citizenship, and the International Regulation of Precarious Employment. New York, United States: Oxford University Press. (2010) p. 1–6.

[7] Kalleberg AL Vallas SP. Probing Precarious Work: Theory, Research, and Politics. In: Precarious work [Internet]. Leeds, England, United Kingdom: Emerald Publishing Limited. (2017) p. 1–30. doi: 10.1108/S0277-283320170000031017

[8] Hsieh YC Apostolopoulos Y Hatzudis K Sonmez S. Occupational exposures and health outcomes among Latina hotel cleaners. Hisp Health Care Int. (2014) 12:6–15. doi: 10.1891/1540-4153.12.1.6, 24865435

[9] Hsieh YC Apostolopoulos Y Sonmez S. Work conditions and health and well-being of Latina hotel housekeepers. J Immigr Minor Health. (2016) 18:568–81. doi: 10.1007/s10903-015-0224-y, 26001842

[10] Sanon MAV. Agency-hired hotel housekeepers: an at-risk group for adverse health outcomes. Workplace Health Saf. (2014) 62:81–5; quiz 86. doi: 10.1177/216507991406200205 24512722 PMC4001253

[11] Rosemberg MS Li Y McConnell DS McCullagh MC Seng JS. Stressors, allostatic load, and health outcomes among women hotel housekeepers: a pilot study. J Occup Environ Hyg. (2019) 16:206–17. doi: 10.1080/15459624.2018.1563303, 30615593 PMC7045341

[12] Bhattacharya A Ray T. Precarious work, job stress, and health-related quality of life. Am J Ind Med. (2021) 64:310–9. doi: 10.1002/ajim.23223, 33543533 PMC9904539

[13] Eggerth DE DeLaney SC Flynn MA Jacobson CJ. Work experiences of Latina immigrants: a qualitative study. J Career Dev. (2012) 39:13–30. doi: 10.1177/0894845311417130, 26346566 PMC4560686

[14] Milczarek-Desai S. Opening the pandemic portal to re-imagine paid sick leave for immigrant workers. Calif Law Rev. (2023) 111:117.

[15] Milczarek-Desai S Sklar T. Immigrant workers’ voices as catalysts for reform in the long-term care industry. Ariz State Law J [Internet]. (2023) 55:891–949.

[16] Krause N Lee PT Scherzer T Rugulies R Sinnott PL Baker RL. Health and working conditions of hotel guest room attendants in Las Vegas. [Internet]. San Francisco, California: University of California at San Francisco. (2002). Available online at: https://lohp.berkeley.edu/wp-content/uploads/2013/10/vegasrpt.pdf

[17] Rosemberg MS Li Y. Effort-reward imbalance and work productivity among hotel housekeeping employees: a pilot study. Workplace Health Saf. (2018) 66:516–21. doi: 10.1177/2165079918755803, 29577838

[18] Rosemberg MA Gultekin L Pardee M. High-ACE low wage workers: occupational health nursing research and praxis through a trauma-informed Lens. Workplace Health Saf. (2018) 66:233–40. doi: 10.1177/2165079917736070, 29168437

[19] Frumin E Moriarty J Vossenas P Punnett L. Workload-related musculoskeletal disorders among hotel housekeepers.[Internet]. (2006). Available online at: https://www.academia.edu/13255563/Workload_Related_Musculoskeletal_Disorders_among_Hotel_Housekeepers_Employer_Records_Reveal_a_Growing_National_Problem

[20] Rosemberg MAS Tsai JHC. Connecting gender, race, class, and immigration status to disease management at the workplace. J Health Disparities Res Pract. (2014) 7:13–31. PMC5044869 27695659

[21] Rosemberg MAS Ghosh B Shaver J Militzer M Seng J McCullagh MC. Blood pressure and job domains among hotel housekeepers. J Health Disparities Res Pract. (2018) 11:101–15.

[22] Sanon MA. Hotel housekeeping work influences on hypertension management. Am J Ind Med. (2013) 56:1402–13. doi: 10.1002/ajim.22209, 23775918 PMC3880392

[23] Krause N Arias O. Disparities in prevalence, treatment, and control of hypertension among low wage immigrant workers beyond health insurance coverage: the Las Vegas hotel room cleaners blood pressure study. J Hypertens Manag. (2015) 1:1–8. doi: 10.23937/2474-3690/1510003

[24] Sabo S Shaw S Ingram M Teufel-Shone N Carvajal S de Zapien JG . Everyday violence, structural racism and mistreatment at the US-Mexico border. Soc Sci Med. (2014) 109:66–74. doi: 10.1016/j.socscimed.2014.02.005, 24705336

[25] Wallerstein N Duran B Oetzel JG Minkler M. Community-based Participatory Research for Health: Advancing Social and Health Equity. San Francisco, California: Jossey-Bass. (2017).

[26] American Public Health Association. Support for community health workers to increase health access and to reduce health inequities [internet]. (2009). Available online at: https://www.apha.org/policies-and-advocacy/public-health-policy-statements/policy-database/2014/07/09/14/19/support-for-community-health-workers-to-increase-health-access-and-to-reduce-health-inequities (Accessed October 16, 2025).

[27] National Institutes of Health. Role of community health workers. (2022). Available online at: https://www.nhlbi.nih.gov/health/educational/healthdisp/role-of-community-health-workers.htm#source1 (Accessed October 16, 2025).

[28] Your heart, your life: community health worker resources for Hispanics/Latinos | NHLBI, NIH. Available online at: https://www.nhlbi.nih.gov/education/heart-truth/CHW/YHYL (Accessed October 21, 2025). (2025)

[29] Nuño T Torres MR Soto S Sepulveda R Aceves B Rosales CB. Feasibility and outcomes of Meta Salud diabetes behavioral health intervention: a pilot study of a community health worker-administered educational intervention to prevent cardiovascular disease and its complications among Hispanic patients with type-2 diabetes. Int J Environ Res Public Health. (2023) 20:6968. doi: 10.3390/ijerph20216968 37947526 PMC10649125

[30] Staten LK Cutshaw CA Davidson C Reinschmidt K Stewart R Roe DJ. Effectiveness of the Pasos Adelante chronic disease prevention and control program in a US-Mexico border community, 2005-2008. Prev Chronic Dis. (2012) 9:100301. doi: 10.5888/pcd9.100301 PMC3277389 22172175

[31] Sabo S Jiménez DJ Longorio AS Gomez O Liebert M Cuautle MA . Aquí entre nos (just between us): engagement of hotel housekeepers during sociopolitical and environmental change. Prog Community Health Partnersh. (2024) 18:213–23. doi: 10.1353/cpr.2024.a930717, 38946566

[32] Dal Grande E Fullerton S Taylor AW. Reliability of self-reported health risk factors and chronic conditions questions collected using the telephone in South Australia, Australia. BMC Med Res Methodol. (2012) 12:108. doi: 10.1186/1471-2288-12-108 22834889 PMC3441283

[33] Swanberg JE Nichols HM Clouser JM Check P Edwards L Bush AM . A systematic review of community health workers’ role in occupational safety and Health Research. J Immigr Minor Health. (2018) 20:1516–31. doi: 10.1007/s10903-018-0711-z, 29502238

[34] Coulter K Ingram M McClelland DJ Lohr A. Positionality of community health workers on health intervention research teams: a scoping review. Front Public Health. (2020) 8:208. doi: 10.3389/fpubh.2020.00208, 32612967 PMC7308474

[35] Rabin R. Community health workers should be worker advocates. New Solutions J. Environ. Occup.Health Policy. (2022) 32:100–5. doi: 10.1177/10482911221107001, 35702044

[36] Belvis F Bolíbar M Benach J Julià M. Precarious employment and chronic stress: do social support networks matter? Int J Environ Res Public Health. (2022) 19. doi: 10.3390/ijerph19031909 PMC8835513 35162929

[37] Killough CM Madaras A Phillips C Hettema J Ceballos V Fuentes JE . Community health worker insights on promoting research engagement with diverse populations. Front Public Health. (2022) 10:959504. doi: 10.3389/fpubh.2022.959504 36711331 PMC9874150

[38] Jiménez DJ Gomez O Meraz R Pollitt AM Evans L Lee N . Community engagement alliance (CEAL) against COVID-19 disparities: academic-community partnership to support workforce capacity building among Arizona community health workers. Front Public Health. (2023) 11:1072808. doi: 10.3389/fpubh.2023.1072808, 36817902 PMC9932528

[39] Right to work | AFL-CIO. (2025) Available online at: https://aflcio.org/issues/right-work (Accessed 21 October, 2025)

[40] Zoorob M. Does 'right to work' imperil the right to health? The effect of labour unions on workplace fatalities. Occup Environ Med. (2018) 75:736–8. doi: 10.1136/oemed-2017-104747, 29898957

[41] Chava S Danis A Hsu A. The economic impact of right-to-work laws: evidence from collective bargaining agreements and corporate policies. J Financ Econ. (2020) 137:451–69. doi: 10.1016/j.jfineco.2020.02.005

[42] Fortin NM Lemieux T Lloyd N. Right-to-work Laws, Unionization, and Wage Setting. Rochester, NY: Social Science Research Network (2022).

[43] Carvajal SC Kibor C McClelland DJ Ingram M de Zapien JG Torres E . Stress and sociocultural factors related to health status among US-Mexico border farmworkers. J Immigr Minor Health. (2014) 16:1176–82. doi: 10.1007/s10903-013-9853-1, 23813347

[44] Rojas Perez OF Silva MA Galvan T Moreno O Venta A Garcini L . Buscando la Calma Dentro de la Tormenta: a brief review of the recent literature on the impact of anti-immigrant rhetoric and policies on stress among Latinx immigrants. Chronic Stress. (2023) 7:24705470231182475. doi: 10.1177/24705470231182475, 37441366 PMC10334021

[45] Vargas ED Sanchez GR Juárez M. The impact of punitive immigrant laws on the health of Latina/o populations. Policy Polit. (2017) 45:312–37. doi: 10.1111/polp.12203, 29200985 PMC5703223

[46] Gómez S O’Leary AO. “On edge all the time”: mixed-status households navigating health care post Arizona’s most stringent anti-immigrant law. Front Public Health. (2018) 6:383. doi: 10.3389/fpubh.2018.00383 30697536 PMC6340969

