# Supplementary material for: Hearts in Action: supporting precarious workers in Arizona, a community-based participatory research approach
Source: Front Public Health. 2026 Feb 26;14:1736253. doi: 10.3389/fpubh.2026.1736253 (PMC12979431; doi:10.3389/fpubh.2026.1736253)
Supplement: Supplementary file 1 [file Table_1.docx]

**Table 1. *Hearts in Action* Intervention Breakdown**

| Hearts in Action Intervention Session Breakdown | |  |
| --- | --- | --- |
| Session No. | **Topic** | |
| 1 | Group introduction and community agreements | |
|  | Introduction to health and labor | |
|  | *Homework: Survey to understand workplace situation* | |
| 2 | Step 1: What is the problem? | |
|  | Step 2: What is the root cause of the problem? | |
|  | *Homework: Reimagine your workplace worksheet* | |
| 3 | Step 3: Reimagine your workplace | |
|  | Step 4: Create an action plan | |
|  | *Homework: Practice the action plan* | |
| 4 | Step 5: Review and revise the progress on the action plan | |
|  | Final group reflection | |

**Table 2. Participant characteristics relative to primary and secondary outcomes at baseline, n=50**

| Characteristic | Total (n=50) | | Self-assessed health (overall) | | Phys. health limited phys. activity | | Difficulty doing everyday activities | | Phys. health or emotional problems limiting activities | | Self-assessed emotional problems | |
| --- | --- | --- | --- | --- | --- | --- | --- | --- | --- | --- | --- | --- |
|  | *n* | *%* | *mean* | *(SD)* | *mean* | *(SD)* | *mean* | *(SD)* | *mean* | *(SD)* | *mean* | *(SD)* |
|  |  |  |  |  |  |  |  |  |  |  |  |  |
| Gender |  |  | *F=* | *4.2** | *F=* | *1.3* | *F=* | *3.3** | *F=* | *5.7*** | *F=* | *2.3* |
| Female | 34 | 68.0% | 3.3 | (0.9) | 2.0 | (1.1) | 2.1 | (1.2) | 2.2 | (1.1) | 2.4 | (1.2) |
| Male | 15 | 30.0% | 2.5 | (1.1) | 1.6 | (1.0) | 1.3 | (0.5) | 1.3 | (0.6) | 1.9 | (1.1) |
| Other | 1 | 2.0% | 4.0 | (0.0) | 3.0 | (0.0) | 3.0 | (0.0) | 4.0 | (0.0) | 4.0 | (0.0) |
|  |  |  |  |  |  |  |  |  |  |  |  |  |
| Age |  |  | *F=* | *1.5* | *F=* | *0.7* | *F=* | *0.3* | *F=* | *0.9* | *F=* | *0.9* |
| <30 years | 7 | 14.0% | 2.1 | (1.2) | 1.7 | (1.3) | 1.6 | (1.1) | 1.4 | (0.8) | 1.7 | (1.0) |
| 30-39 years | 6 | 12.0% | 3.2 | (0.8) | 1.7 | (1.0) | 1.8 | (0.8) | 2.3 | (1.2) | 2.0 | (1.1) |
| 40-49 years | 22 | 44.0% | 3.3 | (1.0) | 1.9 | (1.1) | 2.0 | (1.1) | 2.0 | (1.0) | 2.5 | (1.2) |
| 50-59 years | 10 | 20.0% | 3.0 | (0.9) | 2.2 | (1.2) | 1.8 | (1.1) | 1.9 | (1.1) | 2.3 | (1.3) |
| 60-69 years | 5 | 10.0% | 3.2 | (0.8) | 2.0 | (1.0) | 1.8 | (1.3) | 2.2 | (1.8) | 2.6 | (1.3) |
|  |  |  |  |  |  |  |  |  |  |  |  |  |
| Racial/ethnic identity |  |  | *F=* | *1.4* | *F=* | *1.1* | *F=* | *1.5* | *F=* | *1.1* | *F=* | *3.1** |
| Hispanic/Latine | 43 | 86.0% | 3.1 | (1.0) | 2.0 | (1.1) | 1.9 | (1.1) | 2.0 | (1.1) | 2.3 | (1.2) |
| White + Hispanic | 2 | 4.0% | 3.5 | (0.7) | 2.5 | (0.7) | 3.0 | (0.0) | 2.5 | (0.7) | 4.0 | (0.0) |
| Other + Hispanic | 2 | 4.0% | 3.5 | (0.7) | 1.5 | (0.7) | 1.5 | (0.7) | 1.5 | (0.7) | 3.0 | (1.4) |
| Prefer not to answer | 3 | 6.0% | 2.0 | (1.0) | 1.0 | (0.0) | 1.0 | (0.0) | 1.0 | (0.0) | 1.0 | (0.0) |
|  |  |  |  |  |  |  |  |  |  |  |  |  |
| Highest level of education |  |  | *F=* | *1.1* | *F=* | *1.9* | *F=* | *1.6* | *F=* | *1.6* | *F=* | *1.0* |
| Less than high school | 22 | 44.0% | 3.0 | (1.1) | 1.6 | (0.9) | 1.6 | (0.8) | 1.7 | (0.9) | 2.2 | (1.2) |
| Some high school | 9 | 18.0% | 2.9 | (0.9) | 1.9 | (1.2) | 1.7 | (1.1) | 2.0 | (1.2) | 2.1 | (1.2) |
| High school | 10 | 20.0% | 3.2 | (0.9) | 2.4 | (1.1) | 2.2 | (1.1) | 2.1 | (1.0) | 2.6 | (1.3) |
| GED | 1 | 2.0% | 4.0 | (0.0) | 4.0 | (0.0) | 4.0 | (0.0) | 4.0 | (0.0) | 4.0 | (0.0) |
| Some university or tech. sch. | 3 | 6.0% | 3.3 | (0.6) | 2.7 | (1.5) | 2.3 | (1.5) | 2.7 | (2.1) | 2.7 | (1.5) |
| 4-year BA/BS | 3 | 6.0% | 3.3 | (1.2) | 1.7 | (1.2) | 2.3 | (1.5) | 2.7 | (0.6) | 2.3 | (0.6) |
| Prefer not to answer | 2 | 4.0% | 1.5 | (0.7) | 1.0 | (0.0) | 1.0 | (0.0) | 1.0 | (0.0) | 1.0 | (0.0) |
|  |  |  |  |  |  |  |  |  |  |  |  |  |
| # adults living in home |  |  | *F=* | *1.2* | *F=* | *3.5** | *F=* | *3.2** | *F=* | *2.3* | *F=* | *3.0* |
| 1 adult | 4 | 8.2% | 3.8 | (0.5) | 3.0 | (0.8) | 3.0 | (0.8) | 3.0 | (1.2) | 3.5 | (1.0) |
| 2 adults | 23 | 46.9% | 2.9 | (1.0) | 2.0 | (1.2) | 1.8 | (1.0) | 1.8 | (1.0) | 2.0 | (1.1) |
| >2 adults | 22 | 44.9% | 3.0 | (1.0) | 1.6 | (0.8) | 1.6 | (1.0) | 1.8 | (1.1) | 2.2 | (1.1) |
|  |  |  |  |  |  |  |  |  |  |  |  |  |
| # children living in home |  |  | *F=* | *0.3* | *F=* | *0.4* | *F=* | *0.6* | *F=* | *0.9* | *F=* | *1.8* |
| 1 child | 11 | 32.4% | 3.2 | (1.0) | 2.1 | (1.3) | 2.1 | (1.4) | 2.5 | (1.4) | 2.8 | (1.3) |
| 2 children | 13 | 38.2% | 3.2 | (0.9) | 1.8 | (1.1) | 1.6 | (0.9) | 1.9 | (1.0) | 2.2 | (1.1) |
| >2 children | 10 | 29.4% | 2.9 | (1.2) | 1.7 | (0.8) | 1.9 | (1.1) | 1.8 | (1.0) | 1.9 | (1.2) |
|  |  |  |  |  |  |  |  |  |  |  |  |  |
| Employment status |  |  | *F=* | *1.1* | *F=* | *1.3* | *F=* | *4.2** | *F=* | *1.5* | *F=* | *0.7* |
| Full time | 22 | 44.0% | 3.0 | (0.9) | 1.7 | (0.9) | 1.5 | (0.6) | 1.7 | (0.8) | 2.1 | (1.1) |
| Part time | 24 | 48.0% | 3.0 | (1.1) | 2.0 | (1.2) | 2.0 | (1.2) | 2.1 | (1.3) | 2.4 | (1.2) |
| Other | 4 | 8.0% | 3.8 | (0.5) | 2.5 | (1.3) | 3.0 | (1.4) | 2.5 | (1.3) | 2.8 | (1.5) |
|  |  |  |  |  |  |  |  |  |  |  |  |  |
| Language spoken at home |  |  | *F=* | *0.6* | *F=* | *0.3* | *F=* | *0.0* | *F=* | *0.4* | *F=* | *0.8* |
| Lang. other than English | 48 | 96.0% | 3.1 | (1.0) | 1.9 | (1.1) | 1.9 | (1.1) | 2.0 | (1.1) | 2.3 | (1.2) |
| English | 2 | 4.0% | 2.5 | (0.7) | 1.5 | (0.7) | 2.0 | (1.4) | 1.5 | (0.7) | 3.0 | (1.4) |
|  |  |  |  |  |  |  |  |  |  |  |  |  |
| Type of health insurance |  |  | *F=* | *1.5* | *F=* | *0.7* | *F=* | *0.0* | *F=* | *0.7* | *F=* | *1.2* |
| Does not have insurance | 35 | 70.0% | 2.9 | (1.0) | 2.0 | (1.2) | 1.9 | (1.1) | 1.9 | (1.1) | 2.2 | (1.2) |
| Private | 1 | 2.0% | 4.0 | (0.0) | 1.0 | (0.0) | 2.0 | (0.0) | 1.0 | (0.0) | 1.0 | (0.0) |
| Medicaid, MA, CHIP, state | 1 | 2.0% | 4.0 | (0.0) | 3.0 | (0.0) | 2.0 | (0.0) | 3.0 | (0.0) | 4.0 | (0.0) |
| Other | 13 | 26.0% | 3.4 | (1.0) | 1.8 | (0.8) | 1.8 | (1.1) | 2.2 | (1.2) | 2.5 | (1.3) |
|  |  |  |  |  |  |  |  |  |  |  |  |  |
| Place of birth |  |  | *F=* | *0.0* | *F=* | *4.0* | *F=* | *0.0* | *F=* | *0.0* | *F=* | *0.4* |
| USA | 1 | 2.0% | 3.0 | (0.0) | 4.0 | (0.0) | 2.0 | (0.0) | 2.0 | (0.0) | 3.0 | (0.0) |
| Outside the USA | 49 | 98.0% | 3.0 | (1.0) | 1.9 | (1.1) | 1.9 | (1.1) | 2.0 | (1.1) | 2.3 | (1.2) |
|  |  |  |  |  |  |  |  |  |  |  |  |  |
| Years in the United States |  |  | *F=* | *1.6* | *F=* | *3.0** | *F=* | *1.3* | *F=* | *1.4* | *F=* | *1.6* |
| 0-9 years | 14 | 28.6% | 2.7 | (1.2) | 1.5 | (0.9) | 1.8 | (1.0) | 1.9 | (1.0) | 1.9 | (1.1) |
| 10-19 years | 9 | 18.4% | 3.1 | (0.9) | 1.8 | (1.0) | 1.6 | (0.7) | 2.0 | (1.1) | 2.6 | (1.2) |
| 20-29 years | 20 | 38.8% | 3.0 | (1.0) | 1.8 | (1.1) | 1.8 | (1.2) | 1.7 | (1.0) | 2.1 | (1.2) |
| ≥30 years | 7 | 14.3% | 3.7 | (0.5) | 2.9 | (0.9) | 2.6 | (1.3) | 2.7 | (1.5) | 3.0 | (1.3) |
|  |  |  |  |  |  |  |  |  |  |  |  |  |
| Occupation |  |  | *F=* | *0.5* | *F=* | *1.2* | *F=* | *1.6* | *F=* | *2.9** | *F=* | *0.9* |
| Agriculture | 16 | 32.0% | 2.8 | (1.1) | 1.6 | (0.8) | 1.3 | (0.5) | 1.4 | (0.7) | 2.0 | (1.2) |
| Landscaping | 3 | 6.0% | 2.3 | (1.2) | 1.7 | (1.2) | 1.0 | (0.0) | 1.3 | (0.6) | 1.7 | (0.6) |
| Hotel housekeeping/ domestic cleaning | 23 | 46.0% | 3.2 | (1.0) | 2.1 | (1.1) | 2.2 | (1.2) | 2.3 | (1.3) | 2.5 | (1.3) |
| Restaurant/food | 7 | 14.0% | 3.3 | (0.8) | 1.9 | (1.2) | 2.3 | (1.1) | 2.1 | (0.9) | 2.3 | (1.0) |
| Construction | 1 | 2.0% | 3.0 | (0.0) | 4.0 | (0.0) | 2.0 | (0.0) | 2.0 | (0.0) | 3.0 | (0.0) |
|  |  |  |  |  |  |  |  |  |  |  |  |  |
| Group (clustering by CHW) |  |  | F= | 3.6** | F= | 2.1 | F= | 2.8* | F= | 4.0** | F= | 3.0* |
| One | 10 | 20.0% | 2.4 | (1.1) | 1.3 | (0.7) | 1.6 | (0.8) | 1.3 | (0.6) | 1.7 | (1.0) |
| Two | 8 | 16.0% | 3.6 | (0.6) | 2.1 | (1.0) | 2.4 | (1.1) | 2.3 | (1.1) | 2.4 | (1.5) |
| Three | 3 | 6.0% | 3.5 | (0.8) | 1.8 | (0.8) | 1.3 | (0.5) | 1.5 | (0.8) | 2.3 | (1.2) |
| Four | 15 | 30.0% | 3.1 | (1.0) | 2.1 | (1.2) | 2.0 | (1.2) | 2.2 | (1.0) | 2.6 | (1.2) |
| Five | 5 | 10.0% | 3.1 | (0.7) | 1.4 | (0.8) | 1.3 | (0.5) | 1.2 | (0.4) | 1.5 | (0.7) |
| Six | 9 | 18.0% | 2.7 | (1.0) | 1.7 | (1.0) | 1.6 | (0.8) | 1.9 | (1.3) | 1.8 | (0.9) |
|  |  |  |  |  |  |  |  |  |  |  |  |  |
| Clinical diagnosis |  |  |  |  |  |  |  |  |  |  |  |  |
| Hypertension | 15 | 30.0% |  |  |  |  |  |  |  |  |  |  |
| Pre-hypertension | 6 | 12.0% |  |  |  |  |  |  |  |  |  |  |
| Cholesterol | 15 | 30.0% |  |  |  |  |  |  |  |  |  |  |
| Diabetes | 6 | 12.0% |  |  |  |  |  |  |  |  |  |  |
| Pre-Diabetes | 13 | 26.0% |  |  |  |  |  |  |  |  |  |  |
| Cardiovascular disease | 3 | 6.0% |  |  |  |  |  |  |  |  |  |  |
| Depression | 13 | 26.0% |  |  |  |  |  |  |  |  |  |  |

*p<0.05

**p<0.01

**Table 3. Mixed effects models for primary and secondary outcomes, post-intervention period***

|  | **Model 1** | | **Model 2** | | **Model 3** | |
| --- | --- | --- | --- | --- | --- | --- |
| **Independent variables** | Beta | (95% CI) | Beta | (95% CI) | Beta | (95% CI) |
| Overall self-assessed health | -0.2 | (-0.4, 0.1) | -0.2 | (-0.4, 0.1) | 0.0 | (-0.4, 0.4) |
| Physical health limiting physical activity | -0.3 | (-0.6, 0.0) | -0.3 | (-0.6, 0.0) | -0.3 | (-0.6, 0.1) |
| Difficulty doing everyday activities | -0.1 | (-0.5, 0.2) | -0.1 | (-0.5, 0.2) | 0.2 | (-0.4, 0.8) |
| Physical health or emotional problems limiting social activities | -0.3 | (-0.5, 0.0) | -0.3 | (-0.5, 0.0) | 0.0 | (-0.5, 0.4) |
| Self-assessed emotional problems | -0.3 | (-0.7, 0.0) | -0.3 | (-0.7, 0.0) | -0.7 | (-1.5, 0.1) |

*** *Values for each of the three models are derived from mixed-effects maximum likelihood regressions. In these models, the post-intervention period and specified covariates are treated as fixed effects, while a random intercept is included for individuals to account for within-subject correlation.*

**Table 4. Tertiary outcome (self-efficacy) relative to baseline and follow-up***

| **Item** | **Baseline** | | **Follow-up** | |
| --- | --- | --- | --- | --- |
|  | Mean | (SD) | Mean | (SD) |
| I remain calm when faced with difficulties at work because I trust my abilities. | 3.5 | (0.6) | 3.7 | (0.6) |
| When I face a problem at work, I usually find several solutions. | 3.4 | (0.8) | 3.6 | (0.7) |
| I can usually handle any problem that comes my way at work. | 3.3 | (0.8) | 3.5 | (0.7) |
| My past experiences at work have prepared me well for my future career. | 3.6 | (0.7) | 3.6 | (0.7) |
| I achieve the goals I set for myself at work. | 3.5 | (0.7) | 3.6 | (0.7) |
| I feel prepared for most of the demands of my job. | 3.5 | (0.8) | 3.6 | (0.6) |

*^*^Outcomes were assessed in response to the questions: “How true is the following for you?” with a response set ranging from 1=not at all true to 4=very true*
